# Osteopontin regulates macrophage activation and osteoclast formation in hypertensive patients with vascular calcification

**DOI:** 10.1038/srep40253

**Published:** 2017-01-16

**Authors:** Qian Ge, Cheng-Chao Ruan, Yu Ma, Xiao-Feng Tang, Qi-Hong Wu, Ji-Guang Wang, Ding-Liang Zhu, Ping-Jin Gao

**Affiliations:** 1 State Key Laboratory of Medical Genomics, Shanghai Key Laboratory of Hypertension and Department of Hypertension, Ruijin Hospital and Shanghai Institute of Hypertension, Shanghai Jiao Tong University School of Medicine, Shanghai, China; 2 Laboratory of Vascular Biology and Key Laboratory of Stem Cell Biology, Institute of Health Sciences, Shanghai Institutes for Biological Sciences, Chinese Academy of Sciences, Shanghai, China

## Abstract

Vascular calcification (VC) is a highly regulated ectopic mineral deposition process involving immune cell infiltration in the vasculatures, which has been recognized to be promoted by hypertension. The matricellular glycoprotein osteopontin (OPN) is strongly induced in myeloid cells as a potential inflammatory mediator of vascular injury. This study aims to examine whether OPN is involved in the regulation of macrophage activation and osteoclast formation in hypertensive subjects with VC. We firstly found an increased proportion of CD11c+CD163- pro-inflammatory peripheral monocytes in hypertensive subjects with VC compared to those without VC by flow cytometric analysis. Primary cultured macrophages from hypertensive subjects with VC also showed altered expression profile of inflammatory factors and higher serum OPN level. Exogenous OPN promoted the differentiation of peripheral monocytes into an alternative, anti-inflammatory phenotype, and inhibited macrophage-to-osteoclast differentiation from these VC patients. In addition, calcified vessels showed increased osteoclasts accumulation accompanied with decreased macrophages infiltration in the of hypertensive subjects. Taken together, these demonstrated that OPN exerts an important role in the monocytes/macrophage phenotypic differentiation from hypertensive patients with VC, which includes reducing inflammatory factor expression and attenuating osteoclast formation.

Vascular calcification (VC) acts as a complex pathological process characterized by deposition of mineral in the vasculatures, which has been recognized to be a chronic inflammatory disease and a predictor of future cardiac events and deaths^1^,^2^,^3^. Hypertension is an independent and strong predictor of vascular calcification^4^, which is accompanied with immune cell infiltration in the blood vessels, including monocytes/macrophages, lymphocytes, granulocytes^5^,^6^. Among these, monocytes/macrophages activation, including pro-inflammatory “classical” activation profile (M1) and anti-inflammatory “alternative” activation profile (M2), was involved in the hypertension-related vascular disease^7^,^8^,^9^. In the process of VC, it is a crucial step that monocytes/macrophages infiltrate into the subendothelial space of large arteries^10^, where macrophages could specifically differentiated into osteoclasts by the combined actions of macrophage-colony stimulating factor (M-CSF) and receptor activator for nuclear factor-κ B ligand (RANKL)^11^. However, it is not clear whether macrophage-mediated inflammation is involved in the pathologic processes of VC in the hypertensive patients.

Osteopontin (OPN) is an integrin-binding ligand, N-linked glycoprotein. OPN could be either protective or detrimental to the vascular injury. For example, OPN-meditated inflammation promotes vascular remodeling in atherosclerotic vascular disease^12^,^13^. Blockade of OPN inhibited macrophage chemotaxis in vascular balloon injury model^14^. In contrast, OPN may possess protective effects against vascular calcification^15^,^16^. In humans, serum OPN level is positively associated with arterial stiffness, and significantly increased in arterial hypertension with up-regulated inflammatory factors^17^,^18^. However, whether OPN affect inflammatory factors expression and peripheral monocytes differentiation in hypertensive patients with VC remains poorly understood. In the present study, we recruited hypertensive patients who were diagnosed with (or without) VC by computed tomography scans of the aorta. We investigated inflammatory factors expression in primary cultured macrophages from hypertensive patients with (or without) VC. We also examined the effects of OPN on inflammatory factors expression and macrophage to osteoclast differentiation. In addition, we assessed the OPN expression, macrophage infiltration and osteoclast deposition in the calcific vessels.

## Results

### Hypertensive patients with or without C

The flow chart involved in samples collection and study process were shown in Figure S1. We recruited 70 subjects with hypertension, male 67.1%, average age was 59.33 ± 7.15 yrs, which included 38 VC samples characterized by abdominal CT scan (Figure S2). We adjusted the traditional cardiovascular risk factors including age, gender, and duration of hypertension. There were no statistically significant differences between VC and NO-VC groups in body mass index (BMI), mean systolic blood pressure of 24-hours ambulatory (24 h-SBP), serum glucose, low density lipoprotein (LDL) cholesterol, triglyceride, cholesterol, creatinine, uric acid, albumin creatinine rate (ACR), estimated glomerular filtration rate (eGFR), sodium, calcium, phosphorus, parathyroid hormone (PTH), 1α, 25-dihydroxyvitamin D3 (VitD3), carotid intima media thickness (IMT), ankle brachial index (ABI). However, brachial ankle pulse wave velocity (baPWV) in hypertensive patients with VC was higher than those without VC (Table 1, Table S1). Besides, the antihypertensive drugs and other specific drugs of patients were showed in Table S2.

### Altered inflammatory factors expression in primary macrophages from hypertensive patients with VC

**Figure 1:**
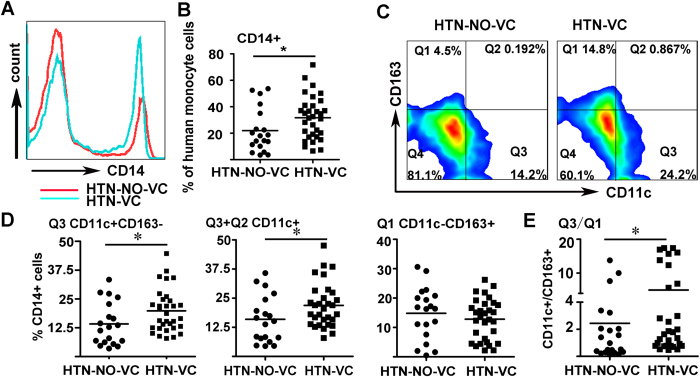
The phenotype of peripheral monocytes from hypertensive patients without vascular calcification (HTN-NO-VC) and with vascular calcification (HTN-VC). (**A**) Peripheral blood mononuclear cells (PBMCs) were labeled with fluorochrome-conjugated antibodies to CD14, CD11c and CD163, flow cytometric analysis shows an increased proposition of CD14+ monocytes in HTN-VC group compared with HTN-VC. (**B**) Quantification of flow cytometric analysis of CD14+ monocytes. *P < 0.05. (**C**) CD14+ monocytes were then analyzed for M1 subtype (CD11c+) and M2 subtype (CD163+). (**D**) Quantification of flow cytometric analysis of CD11c+CD163- (Q3), CD11c+ (Q3 + Q2), CD11c-CD163+ (Q1) and the rate of Q3/Q1 (n = 20–38). *P < 0.05.

To determine the immune phenotype of peripheral monocytes in the hypertensive patients with VC, we firstly detected the surface markers of monocytes by flow cytometry. The results showed a significant increase of CD14+ cells in hypertensive patients with VC compared to those without VC (*P* < 0.05; Fig. 1A,B). In addition, the proportion of CD11c+CD163- pro-inflammatory M1 monocytes was increased in VC group (*P* < 0.05), while the proportion of CD11c-CD163+ anti-inflammatory M2 monocytes had no significant difference between the two groups (*P* > 0.05). The ratio of CD11c+CD163- cells to CD11c-CD163+ cells in hypertensive subjects with VC was higher than those without VC (*P*  <  0.05; Fig. 1C,D).

**Figure 2:**
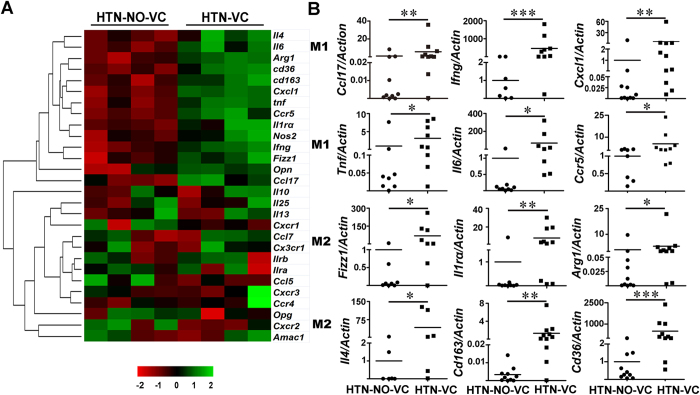
Inflammatory factors expression in primary cultured macrophages from hypertensive patients without vascular calcification (HTN-NO-VC) and with vascular calcification (HTN-VC). (**A**) Heatmap of hierarchical cluster analysis of inflammatory genes expression measured by quantitative reverse transcription polymerase chain reaction (qPCR). (**B**) Representative pro-inflammatory M1 and anti-inflammatory M2 genes were measured by qPCR. Statistically significant differences are indicated by Mann-Whitney U test. *P < 0.05, **P < 0.01, ***P < 0.001 (n = 6–13).

We next detected the inflammatory genes expression in primary cultured macrophages from CD14+ monocytes by qPCR. Cluster analysis of inflammatory genes clearly showed that both pro-inflammatory and anti-inflammatory factor expression were increased in hypertensive subjects with VC (Fig. 2A) compared with those without VC. We then performed confirmative quantitative analysis for several typical inflammatory factors. As shown in Fig. 2B, the M1 pro-inflammatory factors including tumor necrosis factor (*Tnf*), chemokine (C-C motif) ligand 17 (*Ccl17*), chemokine (C-X-C motif) ligand1 (*Cxcl1*), C-C chemokine receptor 5 (*Ccr5*), interferon (*Infg*) and interleukin 6 (*Il6*) and the M2 anti-inflammatory factors including found in inflammatory zone 1 (*Fizz1*), arginase 1 (*Arg1*), interleukin-1 receptor (*Il1ra*), interleukin 4 (*Il4*), *Cd163* and *Cd36* were increased in the VC group. These results suggest a highly active phenotype of peripheral monocytes/macrophages in hypertensive patients with VC.

### OPN regulates inflammatory molecule expression in cultured macrophages from hypertensive patients with VC

**Figure 3:**
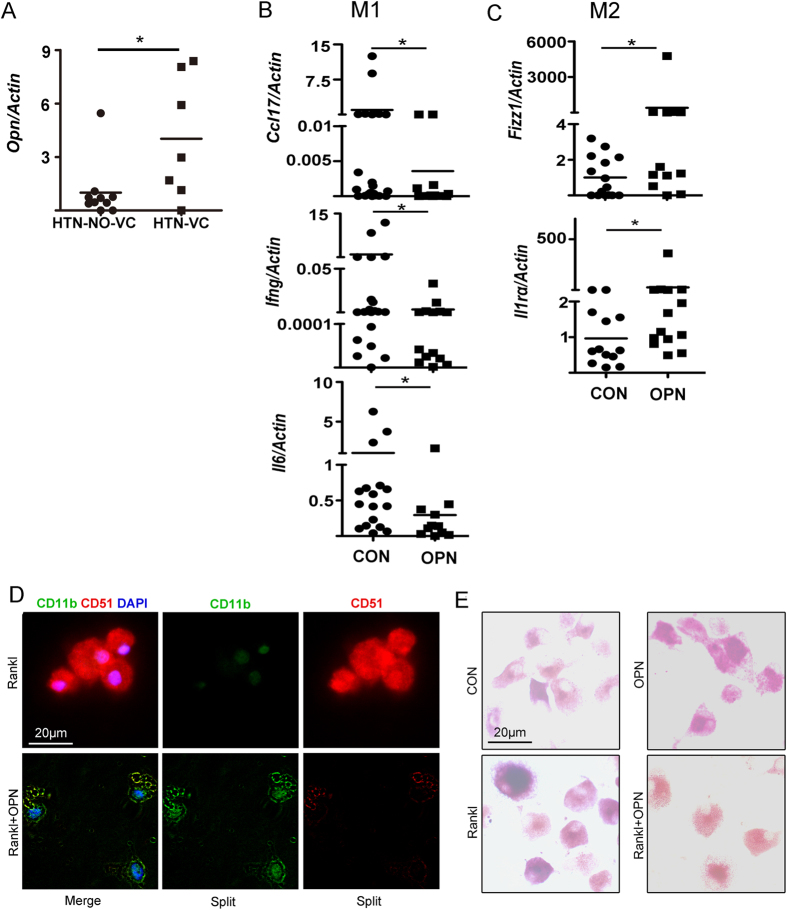
OPN regulates macrophage inflammatory factors expression and macrophage-to-osteoclast differentiation from hypertensive patients with vascular calcification. (**A**) OPN mRNA expressions in human peripheral monocytes-derived macrophages was determined by quantitative reverse transcription polymerase chain reaction (qPCR). The mRNA levels were normalized using β-actin housekeeping gene. *P < 0.05 (n = 7–10). (**B**,**C**) Cultured macrophages were stimulated with OPN (0.5 μmol/l) for 24 hours. Expression of pro-inflammatory M1 and anti-inflammatory M2 genes were measured by qPCR. Statistically significant differences are indicated by Mann-Whitney U test. CON: control. *P < 0.05 (n = 8–15). (**D**) OPN (0.5 μmol/l) pretreated macrophages from hypertensive patients with vascular calcification were induced with Receptor Activator for Nuclear factor-κ B ligand (Rankl 100 ng/mL) for 24 hours. Immunostaining of CD11b (macrophage marker) and CD51 (osteoclast precursor marker). 4′, 6-Diamidino-2-phenylindole (DAPI) was used to detect nucleus (n = 5). Bar: 20 μm. (**E**) OPN treated macrophages from hypertensive patients with vascular calcification were induced with Rankl for 7 days and then were stained by tartrate-resistant acid phosphatase (TRAP) staining (n = 5). Bar: 20 μm.

Next, we detected the OPN expression in the macrophages by qPCR. We found that OPN mRNA level was higher in calcification group than without VC (Fig. 3A, *P* < 0.05). In addition, hypertensive patients with VC had higher serum OPN level than those without VC (*P* < 0.05), and OPN level had positive correlation to baPWV and ABI (*P* < 0.05, Figure S3).

In order to evaluate whether OPN directly affects macrophage activation, the primary cultured macrophages from hypertensive patients were treated with OPN for 24 hours. OPN had no significant effect on macrophages from hypertensive subjects without VC (*P* > 0.05; Figure S4), while in calcific subjects, OPN significantly attenuated pro-inflammatoryfactors expression, including *Ccl17, Infg* and *Il6*, and enhanced anti-inflammatory factors expression, including *Fizz1* and *Il1ra (P* < 0.05; Fig. 3B,C). These results suggest a potential role of OPN-mediated macrophage activation in the regulation of hypertension-related VC.

### OPN inhibits macrophage-to-osteoclast differentiation from hypertensive patients with VC

Next we determined the effect of OPN on macrophage-to-osteoclast differentiation in hypertensive patients with VC. We found that OPN inhibited osteoclast precursor marker CD51 expression and sustained macrophage marker CD11b expression (Fig. 3D). TRAP staining showed that OPN treatment (0.5 μmol/l) inhibited RANKL-induced osteoclast formation from peripheral monocytes of hypertensive patients with VC (Fig. 3E). These results suggest that OPN inhibited macrophage-to-osteoclast differentiation in hypertensive patients with VC.

### Macrophage infiltration and osteoclast accumulation in human calcific vessels

**Figure 4:**
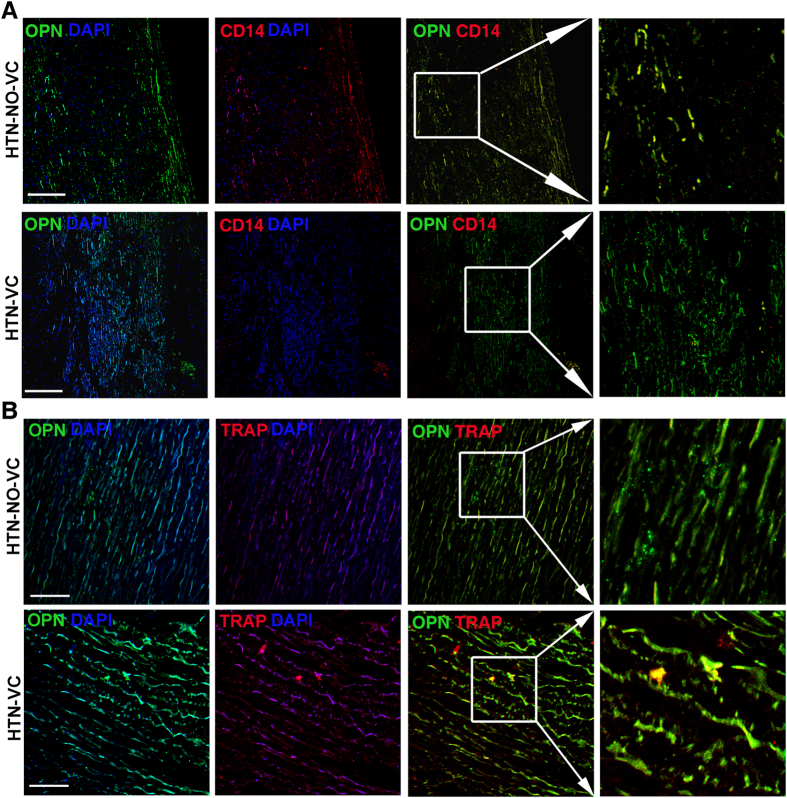
Histological analysis of calcific vessels. Representative immunostaining of OPN and CD14 (**A**), OPN and tartrate-resistant acid phosphatase (TRAP) (**B**) on blood vessels from hypertensive patients without vascular calcification (HTN-NO-VC) and with vascular calcification (n = 5 per group). CD14 represents macrophages, TRAP represents osteoclasts. DAPI (4′, 6-diamidino-2-phenylindole) was used to detect nucleus Bar: 100 μm.

Previous animal studies revealed that vascular macrophage infiltration and osteoclast accumulation are associated with the development of vascular calcification. Next we found that calcified arteries showed positive Alizarin red staining and decreased SM22α positive vascular smooth muscle cells (VSMCs) (*P* < 0.05; Figure S5). In accordance with serum OPN level, calcific vessels showed an increased OPN deposition. There was almost no CD14 positive macrophage infiltration in the calcific vessels, while no-calcific vessels from hypertensive patients showed abundant macrophage infiltration in the vasculatures (Fig. 4A). In addition, we detected an increased expression of osteoclast phenotypic marker TRAP in the calcific vessels (Fig. 4B), suggesting the possibility of osteoclast accumulation. These results demonstrated that the reduction of macrophage infiltration was associated increased osteoclast differentiation in the VC processes.

## Discussion

In the present study, all recruited hypertensive subjects underwent CT scanning of abdominal aorta to verify VC. Hypertensive patients with VC show augmented serum OPN level and increased proportion of peripheral pro-inflammatory monocytes compared to hypertensive patients without VC. Most important, our finding showed that OPN regulates monocyte/macrophage phenotypic differentiation in the hypertensive patients with VC, including inhibition of inflammatory factors expression and attenuation of macrophage-to-osteoclast differentiation.

Chronic inflammation has been well documented in animal VC models^1^,^19^. Monocytes/Macrophages, including pro-inflammatory M1 and anti-inflammatory M2 subsets play prominent roles in the hypertension-induced arterial stiffness, target organ damage, morbidity and mortality^5^,^20^. Recent research showed that alternatively-activated mouse M2 macrophages inhibits calcium-phosphate deposition through increased accumulation of extracellular ATP and pyrophosphate^21^. Clinical study has revealed that isolated systolic hypertension may be relevant in diagnosing or preventing calcified atherosclerosis^22^. The pathological process of VC involves monocyte infiltration and macrophage accumulation in the blood vessels^23^. Histological analysis shows an increased macrophage accumulation in the calcified regions^24^. Herein, in accordance with previous animal research, we firstly demonstrated an increased proportion of pro-inflammatory M1 monocytes in the PBMC of hypertensive patients with VC OPN has been well documented as a protective factor against VC pathological process via inhibiting calcium deposition in the VSMCs^15^,^25^. On the other hand, OPN mainly promotes immune cell-mediated inflammation and accelerates vascular remodeling process in the other vascular disease^12^,^26^. Interestingly, we here showed that OPN attenuates inflammatory factors expression in the primary cultured macrophages from hypertensive patients with VC. It is well established that OPN is multifunctional mediator in the cardiovascular disease^12^,^27^. In fact, OPN is a compound arrangement of multiple peptides that includes splice variants and several active proteolytic cleavage products^28^. The full length OPN can be cleaved by thrombin and matrix metalloproteinases (MMPs)^29^. The N-terminal fragment is probably the main pro-inflammatory mediator, while the C-terminal fragment is mainly responsible for the anti-inflammatory role of OPN^30^,^31^. Taken together, we speculated a potential compensatory role of OPN in the vascular calcification processes. The Increased OPN promoted MMPs production by macrophages and then resulted in OPN cleavage, which may attenuate inflammation through mediating M2 macrophages polarization.

It is known that calcification in the vascular walls is regulated by both osteoblasts that deposit bone matrix, and osteoclasts that are responsible for matrix resorption^32^. Osteoblasts are primarily derived from the VSMCs^33^,^34^, while osteoclasts are derived from monocytes/macrophages in response to cytokines including M-CSF and RANKL^35^. Previous studies have demonstrated that OPN could inhibit VSMC to osteoblast differentiation and enhanced osteoclast survival^16^,^36^. In this study, in accordance with serum OPN level, OPN deposition is increased in the calcific vessels. We found an increased osteoclast accumulation in the calcific vessels, which was accompanied with the reduced macrophage infiltration. This might be explained by the macrophage to osteoclast differentiation in the process of VC, and supported by a previous study in animal vascular calcification model^37^. Most interestingly, our study showed that OPN inhibits osteoclast differentiation and reserves macrophage phenotypic marker expression in the peripheral monocytes from hypertensive patients with VC. We speculated that hypertension could induce macrophage accumulation and OPN production in the blood vessels, where basic calcium phosphate crystals can interact with and activate macrophages, inducing a M1 proinflammatory state^38^. To dampen inflammation, activated macrophages may release MMPs, which could cleave the full length OPN. Then the OPN C-terminal induces M1 to M2 macrophage polarization and inhibits macrophage to osteoclast formation. Taken together, OPN is capable of decreasing inflammatory factor expression and shifting macrophage to osteoclast differentiation into an earlier time point in the pathologic mineralization process of VC in hypertensive patients.

In conclusion, our data showed that an increased serum OPN level is involved in the monocytes/macrophage activation in the hypertensive patients with VC. It appears that OPN antagonizes the macrophage-mediated pathologic mineralization process via two mechanisms: (1) OPN inhibits osteoclast formation from macrophage and (2) OPN attenuates macrophage-derived inflammatory molecule expression. However, the relationship between these two paradox roles of OPN on macrophages from hypertensive patients with VC, and the molecular mechanisms of OPN in these pathogenic processes need to be further investigated in the future. Notwithstanding this limitation, our study demonstrated an important role of OPN in the regulation of inflammation-mediated VC process in hypertensive patients.

## Material and Methods

### Samples and Diagnostic Criteria

We recruited subjects who were excluded from analysis due to incomplete data, autoimmune, neoplastic and infectious diseases. They were consecutively enrolled from 2013 to 2014 in the Department of Hypertension (Ruijin Hospital Shanghai Jiao Tong University School of Medicine). Hypertension was defined as a systolic blood pressure of 140 mmHg and/or a diastolic blood pressure of 90 mmHg on repeated measurements or receiving antihypertensive treatment^39^. This study was approved by the ethics committee of Shanghai Jiao Tong University School of Medicine, and all the procedures were conformed to the Declaration of Helsinki (2013). ALL patients were informed of the purpose of this study and gave their written consent forms before enrollment.

### Abdominal aorta computed tomography (CT) imaging

70 hypertensive cases underwent 64-multi-detector CT (Discovery CT750 HD, GE Healthcare, Milwaukee, WI, USA; Light Speed VCT, GE). Scans of the abdominal aorta from 1 cm above the origin of the celiac axis to 1 cm below the iliac bifurcation (Figure S2). Detector collimation was 0.625 mm, and axial images were reconstructed with slice thickness of 2.5 mm. The number of voxels ≥130 Hounsfield Unit (HU) within the wall of the aorta was determined as vascular calcification, as described previously^40^,^41^.

### Blood pressure and assessment of target organ damage

We programmed validated oscillometric SpaceLabs 90217 monitors (SpaceLabs, Redmond, WA) to obtain blood pressure readings at 20-minute intervals during daytime (06:00–22:00) and at 30-minute intervals during nighttime (22:00–06:00). All recordings covered >20 hours and included ≥80% of the programmed readings. Two experienced observers measured the carotid intima-media thickness, which was used as the mean value of the right and left sides, according to a Phillips IE33 device (Phillips, Eindhoven, The Netherlands) interfaced with 2.5-MHz phased array probe. Carotid wall thickening is identified as IMT > 0.9 mm or plaque. Venous blood samples, collected after overnight fasting, were analyzed by automated enzymatic method for serum cholesterol, creatinine and uric acid and glucose, etc. GFR is estimated by use of MDRD formula as previously described^42^. A first morning urine sample was collected for the measurement of the urinary albumin (in milligram) and creatinine (in millimoles) concentrations. Microalbuminuria is defined as 24-hours urinary albumin between 30 to 300 mg or albumin-creatinine ratio > 3.5.

### Enzyme-linked immunosorbent assay (ELISA)

Patient serum specimens were stored at −20 °C until analyzed by ELISA for OPN. Samples were assayed according to the manufacturer’s instructions (R&D Systems)^43^,^44^.

### Flow cytometric analysis

Peripheral blood mononuclear cells (PBMCs) were isolated from patients by Ficoll-Paque PLUS (GE Healthcare, Munich, Germany). Single-cells suspensions were prepared as previously described^45^. Cells were washed twice with flow cytometry wash buffer. The fluorochrome-coupled antibodies were applied: anti-CD14, anti-CD11c and anti-CD163 (both from BD Biosciences, Heidelberg, Germany). Samples were assessed by flow cytometry using a BD FACS Calibur device and analyzed by FlowJo software.

### Cell culture and quantitative reverse transcription polymerase chain reaction (qPCR) analysis

The PBMCs were collected as previously described^46^. The primary cultured macrophages were differentiated from PBMCs in the presence of RPMI 1640 medium supplemented with 10% foetal bovine serum (Gibco, Carlsbad, CA, USA) and 10% L929 cell culture supernatants, L-glutamine (2 mM), penicillin (10 U/ml) and streptomycin (10 mg/ml) (all from PAA Laboratories, Cölbe, Germany) for 7 days. The cells were induced by OPN (0.5 μmol/l) (Peprotech, USA)^47^. Total RNA was extracted from cultured macrophages using TRIzol (Invitrogen) according to the manufacturer’s protocol and further processed to obtain cDNA using Transcriptor First Strand cDNA Synthesis Kit (Roche, Mannheim, Germany). The cDNA was subjected to the qPCR analysis. Signals were detected on an ABI Prism Viia 7 machine (Applied Biosystems). Human β-actin was used to normalize the data. Reactions were done at 95 °C for 10 min followed by 40 cycles of 95 °C for 10 sec, 60 °C for 30 sec. Sequences of primers used in this study are provided in Online Table S2. A hierarchical clustering of samples was performed using Cluster 3.0 software (http://bonsai.hgc.jp/~mdehoon/software/cluster/software.htm). Heat maps were then generated by using R-project (http://www.r-project.org).

### Osteoclast Formation

PBMCs, as previously described, were harvested and cultured with M-CSF (50 ng/mL) and RANKL (R&D Systems, 100 ng/mL) for 7–10 days. The culture medium was exchanged with fresh medium every 2 days, and osteoclast formation was evaluated. TRAP staining was performed with the TRAP assay kit (Sigma Aldrich) according the manufacturer. Cells were fixed with 3.7% formaldehyde for 30 sec and incubated for 1 hr at 37 °C, with protection from light in a mixture of fast Garnet GBC, sodium nitrite, naphthol AS-BI phosphoric acid, acetate, and the tartrate of the leukocyte acid phosphatase.

### Histological analysis

The paraffin sections of normal or calcified arteries from patients who underwent coronary artery bypass grafting in the cardiac surgery were performed with Alizarin red staining. Morphometric analysis was performed using Image-Pro Plus software to assess calcific region. Immunofluorescence confocal microscopy was performed as previous described^48^. Primary antibodies used were as followings: OPN (BD Biosciences), SM22α (Abcam), CD14 (BD Biosciences), tartrate-resistant acid phosphatase (TRAP, Santa Cruz). The results were analyzed using a laser scanning confocal microscope system (Zeiss, LSM 710, Germany).

### Statistical analysis

Statistical analysis was carried out using SPSS 19.0 (SPSS) and the GraphPad PRISM software (V.5.00 for Windows; GraphPad Software, San Diego, California, USA); P values < 0.05 were considered significant. Categorical variables were compared with Chi-square test. Normally distributed continuous variables were presented as mean ± SD and analyzed using Student t-test. Skewed variables were analyzed using Mann-Whitney U test.

### Statement

All methods were performed in accordance with the relevant guidelines and regulations. This study was approved by the Committee of Ruijin Hospital, Shanghai Jiaotong University School of Medicine. Furthermore, all subjects provided written informed consent.

## Additional Information

**How to cite this article**: Ge, Q. *et al*. Osteopontin regulates macrophage activation and osteoclast formation in hypertensive patients with vascular calcification. *Sci. Rep.* **7**, 40253; doi: 10.1038/srep40253 (2017).

**Publisher's note:** Springer Nature remains neutral with regard to jurisdictional claims in published maps and institutional affiliations.

## Supplementary Material

Supplementary Data

## Figures and Tables

**Table 1:** Characteristics Samples of HTN With and Without VC.

Variable	HTN-NO-VC (N = 32)	HTN-VC (N = 38)	*P*
Age, yrs	56.33 ± 8.97	60.64 ± 5.42	0.07
Males, %	75.0	60.5	0.2
Duration of hypertension, y	13.65 ± 11.55	21.14 ± 12.14	0.07
BMI, kg/m^2^	26.30 ± 4.31	26.77 ± 2.61	0.6
24 h-SBP, mm Hg	128.06 ± 12.69	135.94 ± 16.63	0.09
24 h-DBP, mm Hg	81.36 ± 8.13	78.08 ± 8.24	0.28
Glucose, mmol/L	6.05 ± 2.71	5.85 ± 0.99	0.8
LDL cholesterol, mmol/L	2.39 ± 0.57	2.86 ± 0.77	0.06
cholesterol, mmol/L	4.23 ± 0.64	4.75 ± 0.87	0.06
HDL cholesterol, mmol/L	1.06 ± ± 0.35	1.05 ± 0.22	0.9
Triglyceride, mmol/L	2.33 ± 1.21	2.32 ± 1.75	1
Creatinine, μmol/L	74.86 ± 16.25	71.25 ± 22.04	0.6
Uric acid, μmol/L	331.93 ± 76.66	366.41 ± 94.36	0.2
U-ACR, mg/mmoL	2.63 ± 1.71	2.55 ± 1.20	0.4
eGFR, ml/min•1.73 m^2^	98.30 ± 15.17	85.33 ± 28.01	0.1

Data was shown as n(%) or mean ± SD; HTN-NO-VC: hypertensive subjects without vascular calcification; HTN-VC: hypertensive subjects with vascular calcification; S: serum; U: urine; 24 h-SBP: mean systolic blood pressure of 24-hours ambulatory blood pressure monitoring; 24 h-DBP: mean diastolic blood pressure; 24-hours ambulatory blood pressure monitoring; LDL: low density lipoprotein; HDL: high density lipoprotein; ACR: albuminecreatinine rate; eGFR: estimated glomerular filtration rate.
