# Supplementary material for: Osteopontin regulates macrophage activation and osteoclast formation in hypertensive patients with vascular calcification
Source: Sci Rep. 2017 Jan 16;7:40253. doi: 10.1038/srep40253 (PMC5238370; doi:10.1038/srep40253)
Supplement: Supplementary Data [file srep40253-s1.doc]

**Supplement information**

**Osteopontin regulates macrophage activation and osteoclast formation in hypertensive patients with vascular calcification**

**Short title:**OPN-mediated macrophage activation in VC

Qian Ge^a,c^, Cheng-Chao Ruan^a,b,c^, Yu Ma^a,b,c^, Xiao-Feng Tang^a^, Qi-Hong Wu^a,c^, Ji-Guang Wang^a,c^, Ding-Liang Zhu^a,c^, and Ping-JinGao^a,b,c^

^a^State Key Laboratory of Medical Genomics, Shanghai Key Laboratory of Hypertension and Department of Hypertension, Ruijin Hospital and Shanghai Institute of Hypertension, Shanghai Jiao Tong University School of Medicine, shanghai, China.

^b^Laboratory of Vascular Biology and Key Laboratory of Stem Cell Biology, Institute of Health Sciences, Shanghai Institutes for Biological Sciences, Chinese Academy of Sciences, Shanghai, China.

^c^Shanghai Institute of Hypertension, Shanghai, China.

**Correspondence authors:**

Ping-Jin Gao MD, PhD, Ruijin Hospital, Shanghai Institute of Hypertension, Shanghai Jiao Tong  University  School  of  Medicine, 197 Ruijin 2nd Rd.Shanghai 200025, China. Tel: +86 21 64370045/610903, Fax: +86 21 64458970, E-mail: gaopingjin@sibs.ac.cn

Cheng-Chao Ruan PhD, Ruijin Hospital, Shanghai Institute of Hypertension, Shanghai Jiao Tong  University  School  of  Medicine, 197 Ruijin 2nd Rd.Shanghai 200025, China. Tel: +86 21 64370045/610905, Fax: +86 21 64458970, E-mail: ccruan@sibs.ac.cn


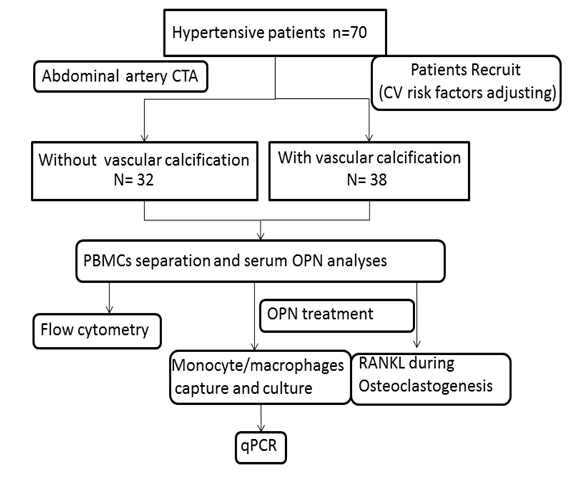


Figure S1 Flow chart of samples collection and study process

CTA：abdominal artery computer tomography, CV risk factors: cardiovascular risk factors, PBMCs: peripheral blood mononuclear cells, OPN: osteopontin


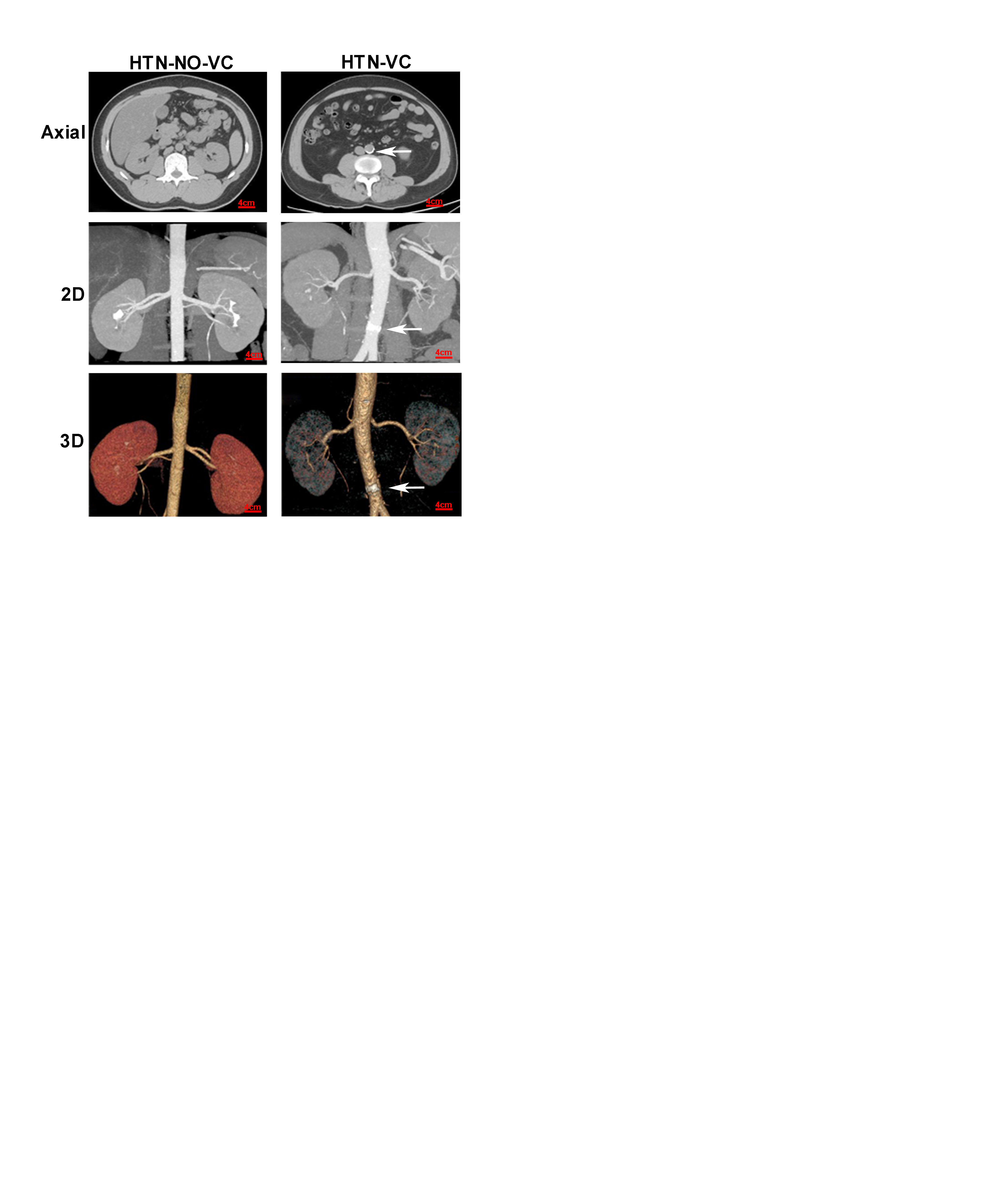


Figure S2 Imaging identification of vascular calcification in the hypertensive patients. Representative abdominal aortic CT images are from hypertensive patients without vascular calcification (HTN-NO-VC) or hypertensive patients with vascular calcification (HTN-VC) by 64-multi-detector CT. The white arrows shows calcific region with attenuation greater than 130-HU level in axial unenhanced images, enhanced maximum intensity projection (MIP) images and volume rendering (VR) images.


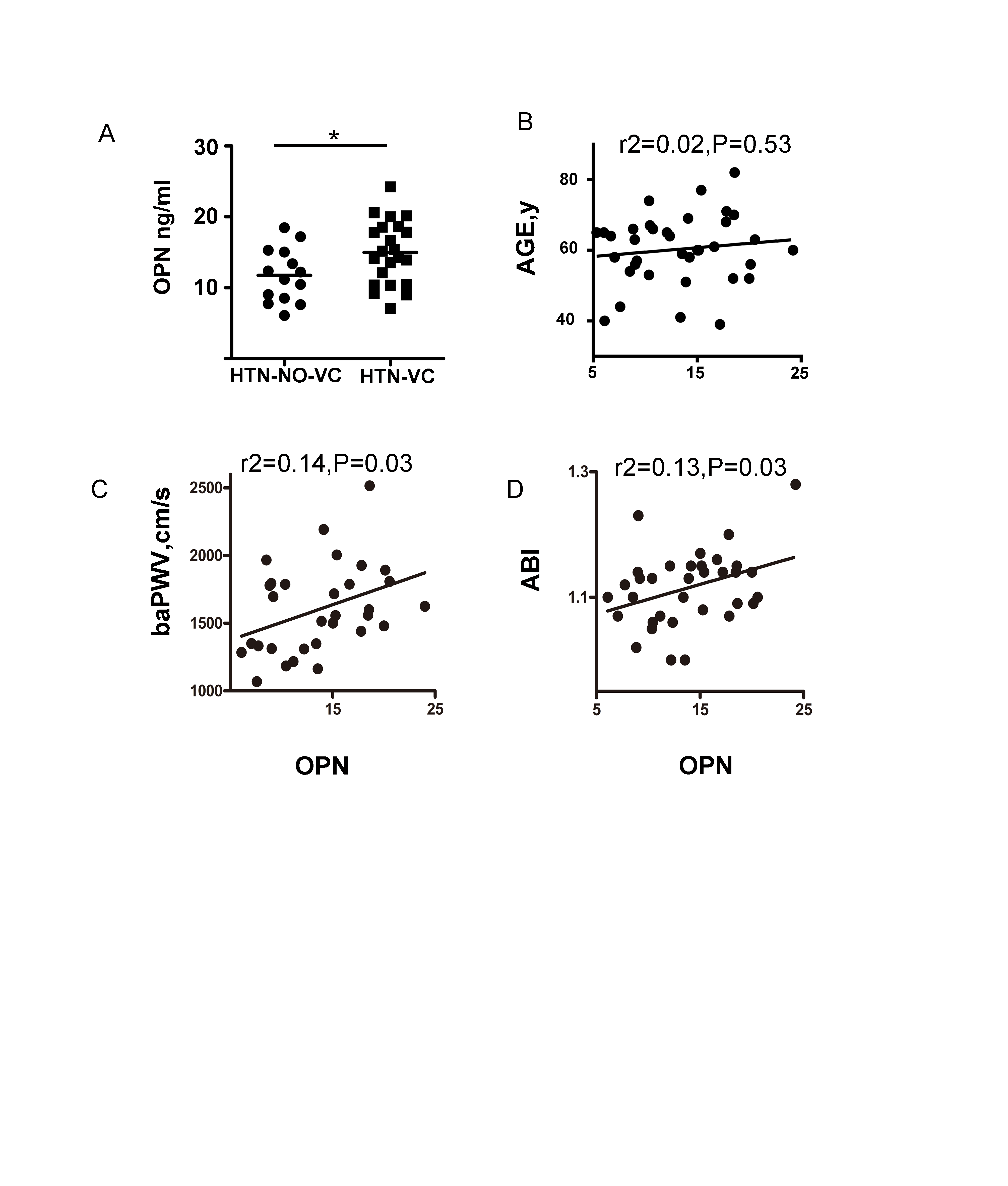


Figure S3 Linear regression analysis of OPN associated with age, baPWV and ABI. baPWV: ankle-brachial pulse wave velocity, ABI: ankle-brachial index. OPN: serum OPN levels, OPG: serum OPN levels, y: years old. Linear regression analysis revealed that serum levels OPN and OPG were associated with abPWV and ABI in hypertension patients. N=41;*P<0.05.


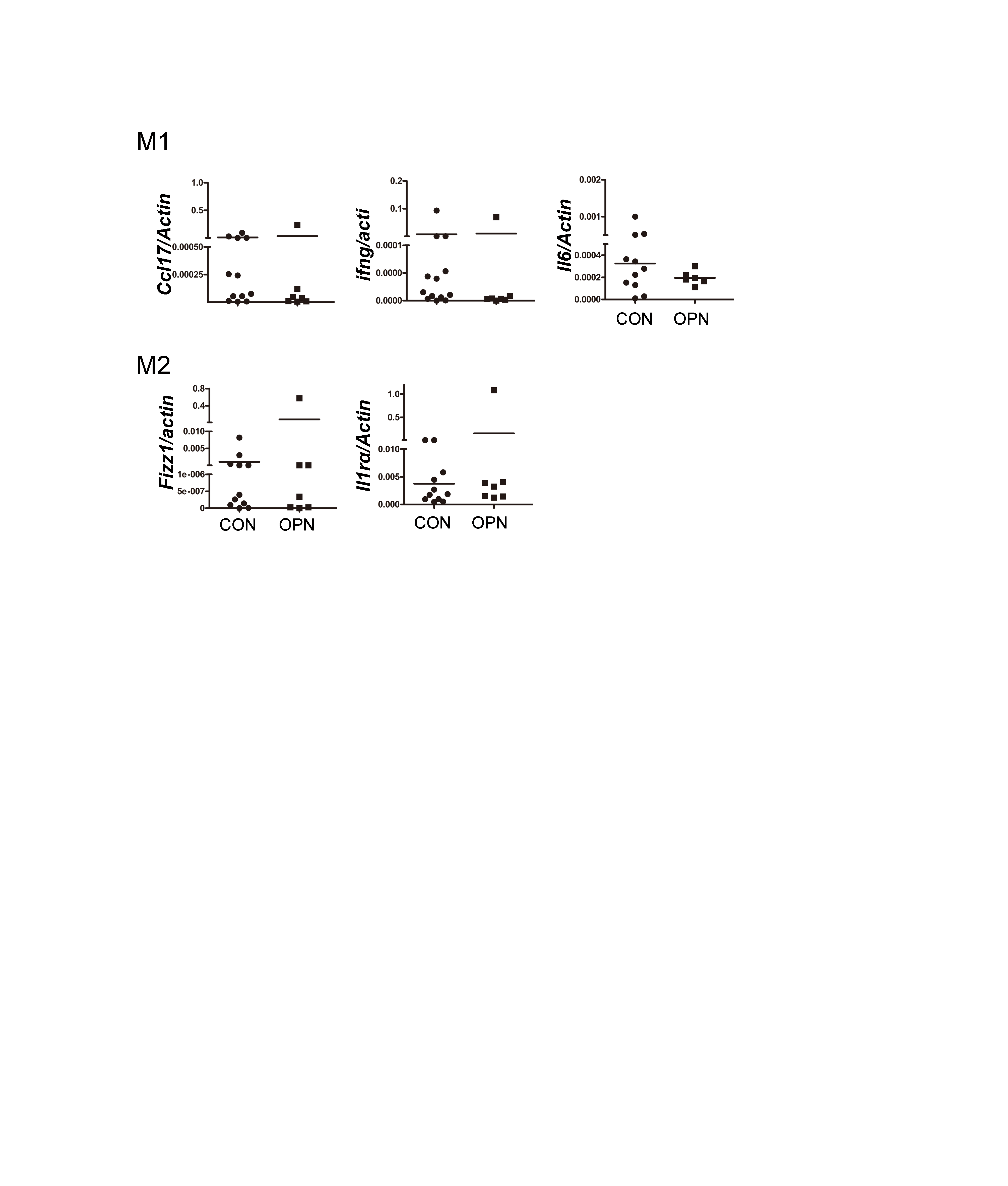


Bar：100μm

Figure S4 M1 and M2 macrophage cytokine genes expression stimulated by OPN in HTN without VC patients.

M1 and M2 macrophage markers are expressed in vitro by incubation of human peripheral blood-derived monocyte cells for 7 days and stimulation with OPN for 24 hours. RNA was extracted from incubated human macrophage cells in vitro in hypertensive patients without VC. Expression of M1 and M2 macrophage cytokine gene levels were measured by qPCR. Statistically significant differences are indicated (Mann-Whitney U test; *p < 0.05, ** p < 0.01, *** p < 0.001, OPN versus control). N=6-13.


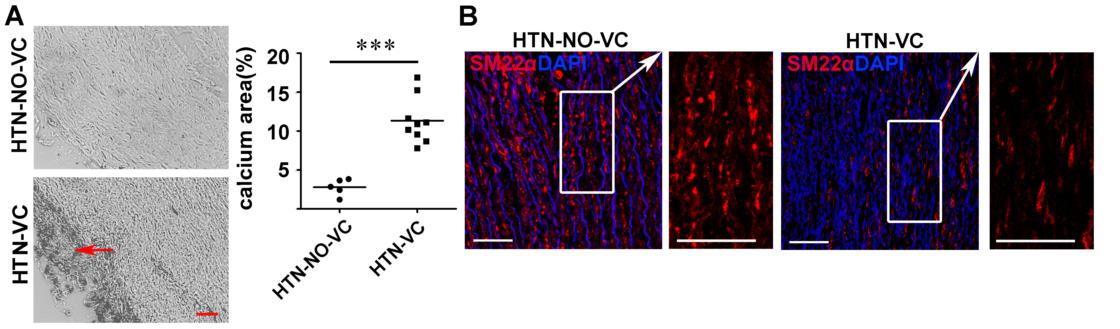


Figure S5 Histological analysis of calcific vessels. (A) Alizarin red staining of the calcific blood vessels (red arrow). Bar: 100 μm. The right panel shows quantitative analysis of calcification in HTN-NO-VC (n=5) and HTN-VC (n=9). *P<0.05. (B) Representative immunostaining of SM22α (vascular smooth muscle cell phenotypic marker) on blood vessels from HTN-NO-VC and HTN-VC patients (n=5 per group). 4’,6-Diamidino-2-phenylindole (DAPI) was used to detect nucleus. Bar: 100 μm.

Table S1. Characteristics Samples of HTN With and Without VC

| Variable | HTN-NO-VC (N=32) | HTN-VC (N=38) | *P* |
| --- | --- | --- | --- |
| S-Phosphorus, mmol/L | 1.09±0.19 | 1.10±0.23 | 0.9 |
| S-Calium, mmol/L | 2.22±0.11 | 2.23±0.09 | 0.6 |
| S-Sodium, mmol/L | 142.14±2.11 | 142.66±1.95 | 0.4 |
| 24hU-Phosphorus, mmol/24h | 16.84±6.39 | 17.64±5.57 | 0.7 |
| 24hU-Calium, mmol/24h | 5.33±2.08 | 5.33±3.99 | 1 |
| 24hU-Sodium, mmol/24h | 172.27±56.03 | 163.72±65.9 | 0.69 |
| S-PTH, pg/mL | 53.87±27.44 | 55.97±24.81 | 0.4 |
| S-VitD3, nmol/L | 49.21±11.46 | 46.67±15.21 | 0.5 |
| IMT, mm | 0.65±0.05 | 0.71±0.09 | 0.7 |
| baPWV, cm/s | 1541.8±316.96 | 1787.70±277.89 | 0.02 |
| ABI | 1.15±0.05 | 1.14±0.07 | 0.8 |

Data was shown as mean±SD; eGFR was estimated by use of MDRD formula; baPWV and ABI are mean value of left and right of samples; IMT, HTN-NO-VC: hypertensive subjects without vascular calcification; HTN-VC: hypertensive subjects with vascular calcification; S: serum; U: urine; eGFR: estimated glomerular filtration rate; PTH: parathyroid hormone; VitD3: 1α,25-dihydroxyvitamin D3; IMT: carotid intima media thickness; baPWV: brachial ankle pulse wave velocity; ABI: ankle brachial index.

Table S2. Medications of HTN With and Without VC

| Medications | | HTN-NO-VC (N=32) | HTN-VC (N=38) | | *P* |  |
| --- | --- | --- | --- | --- | --- | --- |
|  | antihypertension agent |  |  |  | | |
|  | antihypertension agent amout | 2.39±1.47 | 3.34±1.19 | 0.01 | | |
|  | ACEI or ARB n(%) | 22(68.8) | 34(89.5) | 0.03 | | |
|  | CCB n(%) | 28(87.5) | 35(92.1) | 0.52 | | |
|  | Diuretic n(%) | 6(18.8) | 20(52.6) | 0.03 | | |
|  | β-blocker n(%) | 6(18.8) | 23(60.5) | <0.001 | | |
|  | Other antihypertension agent n(%) | 8(25.0) | 15(39.5) | 0.2 | | |
|  | Statin n(%) | 2(6.3) | 8(21.1) | 0.08 | | |
|  | Aspirin n(%) | 1(3.1) | 6(15.8) | 0.08 | | |
|  | Antidiabetic oral agent n(%) | 2(6.3) | 15(39.5) | 0.001 | | |
|  | Insulin n(%) | 1(3.1) | 2(5.3) | 0.66 | | |

Data was shown as n(%) or mean±SD; HTN-NO-VC: hypertensive subjects without vascular calcification; HTN-VC: hypertensive subjects with vascular calcification; ACEI: Angiotensin-Converting Enzyme Inhibitors; ARB: Angiotensin receptor blocker; CCB: Calcium ion antagonist.

Table S3 Chromatin immunoprecipitation PCR primers

|  | Forward primer sequence | Reverse primer sequence |
| --- | --- | --- |
| CCR5 | 5' GGT GTA AAA GGA TGG GTC T | 5' CTG GGA TGG GAA GGA AAT C |
| IL-4 | 5' TGG AAG AGA GGT GCT GAT T | 5' AAC CGA GGG AAA ATG AGT |
| IFNG | 5' CCA ACG CAA AGC AAT ACA T | 5' CAG GCA GGA CAA CCA TTA C |
| CXCL1 | 5' GAA GTC ATA GCC ACA CTC A | 5' GAT TTG TCA CTG TTC AGC A |
| CCL17 | 5' ACC CCA ACA ACA AGA GAG T | 5' GGT GAG GAG GCT TCA AGA C |
| IL1R1 | 5' TGC CCT CTC TGA ATG TTT G | 5' ACT AAG CCA TCT GTA GAC G |
| ARG1 | 5' GAA GAC ACC AGA AGA AGT A | 5' GGT AGT CAA TAG GCT TGT G |
| IL-6 | 5' AAG CCA GAG CTG TGC AGA TGA GTA | 5' TGT CCT GCA GCC ACT GGT TC |
| CD 163 | 5' TTG CCA GCA GT TAA ATG TG | 5' AGG ACA GTG TTT GGG ACT GG |
| Fizz1 | 5' CCC CAG GAC ACT GAC TCT G | 5' GAT TAG GAT GAG AAG GAG G |
| TNF | 5' CAG AGG GCC TGT ACC TCA TC | 5' GGA AGA CCC CTC CCA GAT AG |
| CD36 | 5' TCA GCA AAT GCA AAG AAG GGA GAC | 5' GGT TGA CCT GCA GCC GTT TTG |
| OPN | 5' CCG ATG TGA TTG ATA GTC A | 5' TTC CTT ACT TTT GGG GTC T |
| βactin | 5' CTC CAT CCT GGC CTC GCT GT | 5' GCT GTC ACC TTC ACC GTT CC |
